# High-performance Platinum-free oxygen reduction reaction and hydrogen oxidation reaction catalyst in polymer electrolyte membrane fuel cell

**DOI:** 10.1038/s41598-018-22001-9

**Published:** 2018-02-26

**Authors:** Priji Chandran, Arpita Ghosh, Sundara Ramaprabhu

**Affiliations:** Alternative Energy and Nanotechnology Laboratory (AENL), Nano-Functional Materials and Technology Centre (NFMTC), Department of Physics, Indian Institute of Technology Madras, Chennai, Tamil Nadu 600036 India

## Abstract

The integration of polymer electrolyte membrane fuel cell (PEMFC) stack into vehicles necessitates the replacement of high-priced platinum (Pt)-based electrocatalyst, which contributes to about 45% of the cost of the stack. The implementation of high-performance and durable Pt metal-free catalyst for both oxygen reduction reaction (ORR) and hydrogen oxidation reaction (HOR) could significantly enable large-scale commercialization of fuel cell–powered vehicles. Towards this goal, a simple, scalable, single-step synthesis method was adopted to develop palladium-cobalt alloy supported on nitrogen-doped reduced graphene oxide (Pd_3_Co/NG) nanocomposite. Rotating ring-disk electrode (RRDE) studies for the electrochemical activity towards ORR indicates that ORR proceeds via nearly four-electron mechanism. Besides, the mass activity of Pd_3_Co/NG shows an enhancement of 1.6 times compared to that of Pd/NG. The full fuel cell measurements were carried out using Pd_3_Co/NG at the anode, cathode in conjunction with Pt/C and simultaneously at both anode and cathode. A maximum power density of 68 mW/cm^2^ is accomplished from the simultaneous use of Pd_3_Co/NG as both anode and cathode electrocatalyst with individual loading of 0.5 mg/cm^2^ at 60 °C without any backpressure. To the best of our knowledge, the present study is the first of its kind of a fully non-Pt based PEM full cell.

## Introduction

In recent years, the rate of depletion of fossil fuel reserves is elevating in a rapid speed due to high demand of energy. Moreover, the combustion of fossil fuel causes emission of harmful gases, which lead to adverse effects on environment. The increase in cost of fossil fuels and the environmental pollution drives to find a clean and sustainable alternative energy source. Fuel cells are one of the best alternative energy sources for power generation. Out of the different type of fuel cells, hydrogen fuelled polymer electrolyte membrane fuel cell (PEMFC) has gathered enormous attention due to its zero pollutant emission, high efficiency, fast start up time and low operating temperature^1,2^. However, the commercialization of PEMFC is hindered due to the high cost of various components of a fuel cell. The catalyst used in PEMFC is the highest contributor to the overall cost of a fuel cell. The commonly used catalyst in PEMFC is platinum (Pt) due to its good catalytic activity, stability to withstand the operating environment and resistance to corrosion^2^. However, the less abundance and high cost of Pt which made researchers to put tremendous effort to find an alternative for Pt without compromising the catalytic performance^3,4^.

Recently vast researches have been done on PEMFC using other platinum group metal (PGMs) catalysts and PGM-free catalysts. Though PGM-free catalysts like transition metal-nitrogen-carbon (TM-N-C) based composite have proved to exhibit good ORR activity, most of the studies have shown ORR activity of TM-N-C using electrochemical half-cell measurements^5^. Even though, some groups has shown single cell measurements with TM-N-C as cathode catalyst, the amount of catalyst loading was very high (2–4 mg/cm^2^) compared to conventional PGM based catalyst^6–8^. Thus among other PGMs, palladium (Pd) has the potential to replace Pt in PEMFC. Pd, which is low-priced and abundant relative to Pt, provides good catalytic performance, thereby reducing the cost of a fuel cell as a whole^9,10^. Further reduction in the cost of the catalyst can be achieved by alloying Pd with less expensive transition metals. During the past few decades, several reports clearly depict that Pd-alloy based electrocatalyst has good catalytic activity^11–14^. For instance, Shao *et al*. reported Pd-Fe/C as a good electrocatalyst with high ORR activity^15^. Similarly, Martinez *et al*. demonstrated the high ORR activity of bimetallic electrocatalyst Pd-Co/C compared to monometallic Pd/C^9^.

The performance of a fuel cell depends not only on the catalyst but also on the support material used. The complete utilization of the catalyst is only possible by choosing a good support material with high surface area, so that it can provide more anchoring sites where the catalyst nanoparticles can be attached. Researchers have already extensively reported carbon nanomaterials like carbon black, carbon nanotubes, graphene, carbon nanofibres as supporting materials for anchoring catalyst nanoparticles in PEMFC^16–20^. Among these carbon materials, graphene has proved as an excellent support material due to various reasons like high surface area, good electrical conductivity and good mechanical and chemical stability in operating environment^21–24^. Moreover, graphene is not likely to have any metallic impurities during the preparation process, which is unavoidable in the case of carbon nanotubes which affects the catalytic performance^25,26^. The graphene structure can be modified with the introduction of heteroatoms to the carbon lattice, which can result in increasing the performance via tuning the electronic properties of pristine graphene. The most commonly used heteroatoms are nitrogen, boron, sulphur and phosphorous. Among the various heteroatoms used for doping, nitrogen has been given more importance due to its same atomic size as carbon with one electron more than that of carbon^27^. When nitrogen is incorporated into graphene, the charge distribution of the carbon atoms gets disturbed. This creates some active regions on the surface of graphene. These active regions act as anchoring sites for the metal nanoparticles to get attached^28^. Qazzazie *et al*. reported that the incorporation of nitrogen into graphene enhances the oxygen reduction reaction performance^29^. Similarly, Qu *et al*. demonstrated nitrogen-doped graphene is a good metal free electrocatalyst in fuel cell^30^. Though, several groups have extensively studied Pd alloy based catalyst on carbon support as an efficient cathode electrocatalyst in PEMFC, nobody has reported as anode as well as cathode electrocatalyst simultaneously.

In the present work, palladium-cobalt alloy supported on nitrogen-doped reduced graphene oxide (Pd_3_Co/NG) has been successfully synthesized in a single-step method. The simultaneous reduction of graphene oxide (GO), palladium chloride and cobalt chloride along with the incorporation of nitrogen has been achieved in a single step. The result indicated successful doping of nitrogen and the uniform dispersion of catalyst alloy nanoparticles over nitrogen-doped reduced graphene oxide. Single cell measurements revealed that the prepared electrocatalyst has both good hydrogen oxidation reaction (HOR) activity and oxygen reduction reaction (ORR) activity.

## Results and Discussions

**Figure 1:**
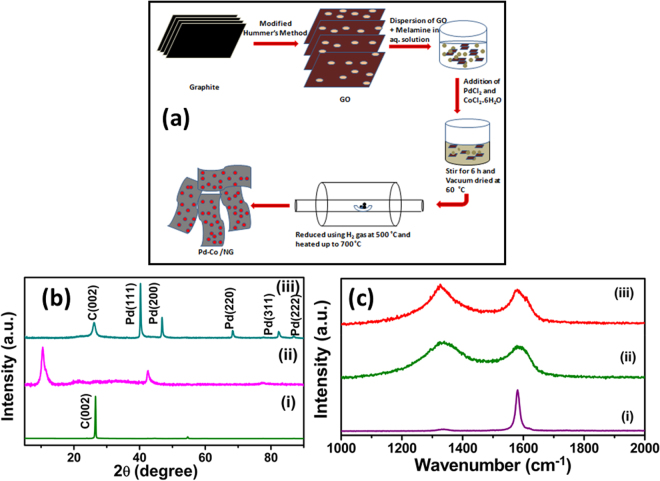
(**a**) Schematic of synthesis of Pd_3_Co/NG, (**b**) XRD pattern of (i) Graphite, (ii) GO, (iii) Pd_3_Co/NG and (**c**) Raman spectra of (i) Graphite, (ii) GO, (iii) Pd_3_Co/NG.

The schematic representation of the synthesized sample Pd_3_Co/NG is shown in the Fig. 1(a). The powder X-ray diffraction (XRD) patterns of graphite, GO, Pd_3_Co/NG are shown in Fig. 1(b). The peak around 26° in Fig. 1(b) (i) corresponds to the characteristic graphitic peak with 0.34 nm d-spacing. In Fig. 1(b) (ii) the peak around 26° shifted to 11° which confirms the formation of GO due to the incorporation of oxygen functional groups in between the layers of graphite which results in increased d-spacing from 0.34 nm to 0.84 nm. Figure 1(b) (iii) shows the XRD of Pd_3_Co/NG. The sharp peak around 11° disappears and a broad peak around 26° confirms the reduction of GO. The peaks around 40°, 46°, 69°, 81°, 86° correspond to Pd (111), Pd (200), Pd (220), Pd (311), Pd (222) thus ensuring the formation of palladium nanoparticles. No separate peaks corresponding to cobalt can be seen in the XRD pattern which confirms the formation of palladium-cobalt alloy^21^. The XRD patterns of Pd_3_Co/NG and palladium supported on nitrogen-doped reduced graphene oxide (Pd/NG) are depicted in Fig. S1 (supporting information) shows that the Pd peaks in the case of Pd_3_Co/NG were observed to shift to higher angles compared to Pd/NG. It is due to the lattice contraction caused by the incorporation of smaller Co atoms in Pd lattice, which further confirms the alloy formation in Pd_3_Co/NG.

Fig. 1(c) illustrates the Raman spectra for (i) graphite, (ii) GO, (iii) Pd_3_Co/NG. There are two prominent peaks in the Raman spectra, D band and G band. The D band gives the degree of disorder present in the carbon material and G band is formed due to the stretching of the C-C bond in the carbon samples which gives the information about the crystalline nature of the material^31^. The ratio between the intensities of D band and G band (I_D_/I_G_) is used to characterize the defects present in the carbon materials. The I_D_/I_G_ ratio of the samples were calculated and listed in the Table S1. Fig. 1(c) (i) shows the Raman spectrum of graphite with sharp G band at 1577.7 cm^−1^ and almost negligible D band around 1328.60 cm^−1^. The intense G band signifies the highly crystalline nature of the graphite and very less defects with I_D_/I_G_ value 0.21. Fig. 1(c) (ii) shows the Raman spectrum of GO with I_D_/I_G_ ratio 1.07, which is high compared to the graphite due to the presence of oxygen containing functional groups in GO. The G band of GO occurs at 1592.45 cm^−1^, higher wave number than the graphite due to the formation of sp^3^ carbon atoms results from the incorporation of oxygen functional groups^32^. In the case of Pd_3_Co/NG (Fig. 1(c) (iii)), the I_D_/I_G_ ratio increased to 1.11, treating palladium and cobalt nanoparticles as defects. In addition to that, nitrogen doping also generates some defects in the system.

**Figure 2:**
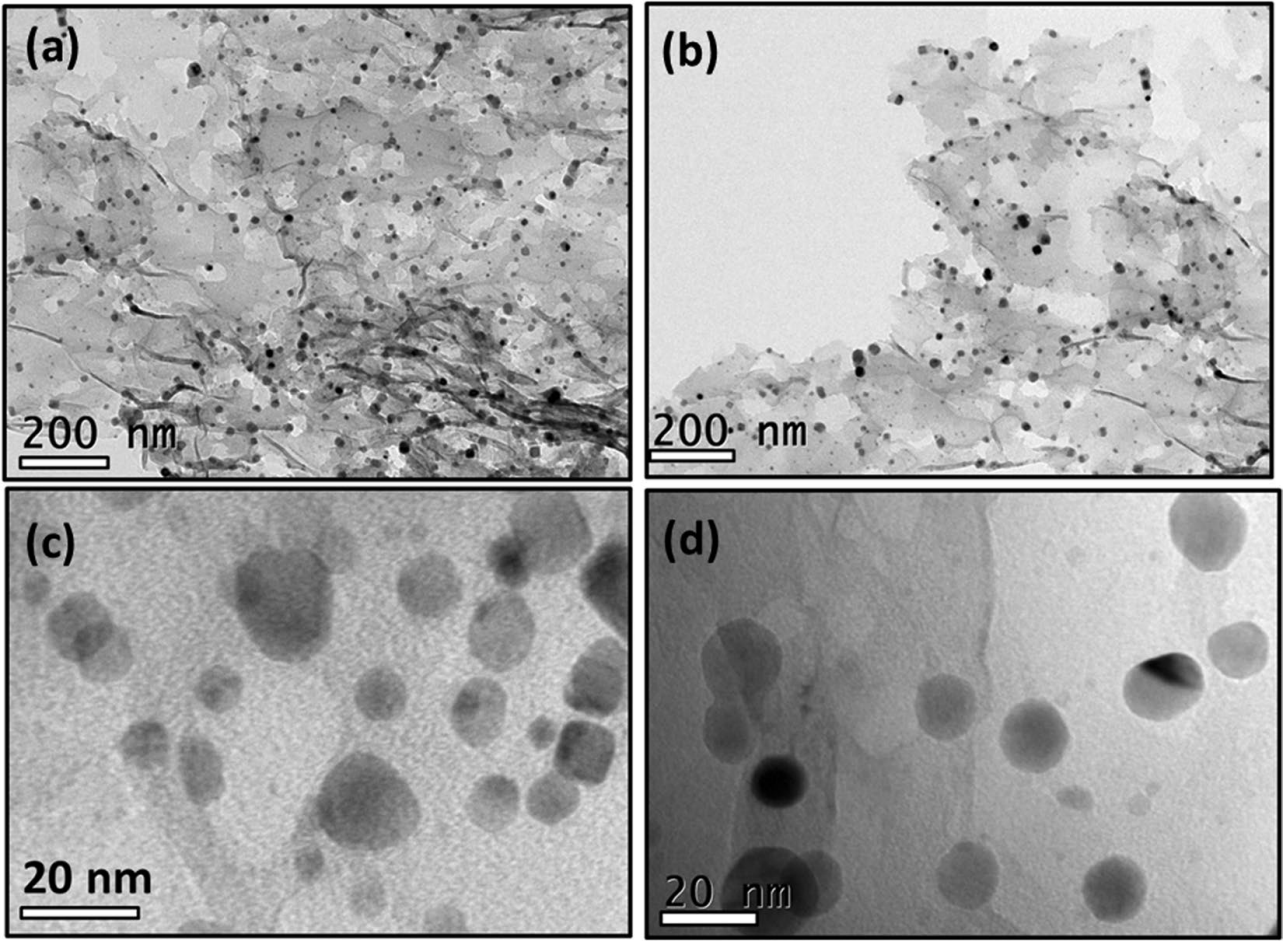
TEM images of (**a**) and (**c**) Pd_3_Co/NG, (**b**) and (**d**) Pd/NG.

The morphological analysis of the samples was studied using scanning electron microscopy (SEM) and transmission electron microscopy (TEM). Fig. S2 shows the SEM image and elemental mapping of the sample Pd_3_Co/NG. The SEM image clearly shows the layered and wrinkled nature of NG and the metal nanoparticles are finely distributed over the surface of the support material NG. To analyse the distribution of the elements present in the sample, the elemental mapping was carried out. The elemental mapping shows a homogeneous distribution of C, N, Pd and Co elements in the sample. Fig. 2(a,c) shows the TEM images of Pd_3_Co/NG and 2 (b,d) of Pd/NG. TEM images show the highly transparent sheet like structure of reduced graphene oxide layers, which signifies the proper exfoliation of GO. A uniform and homogeneous distribution of catalyst nanoparticles over the surface of NG without any agglomeration can be observed. The average particle size was found to be around 20 nm.

**Figure 3:**
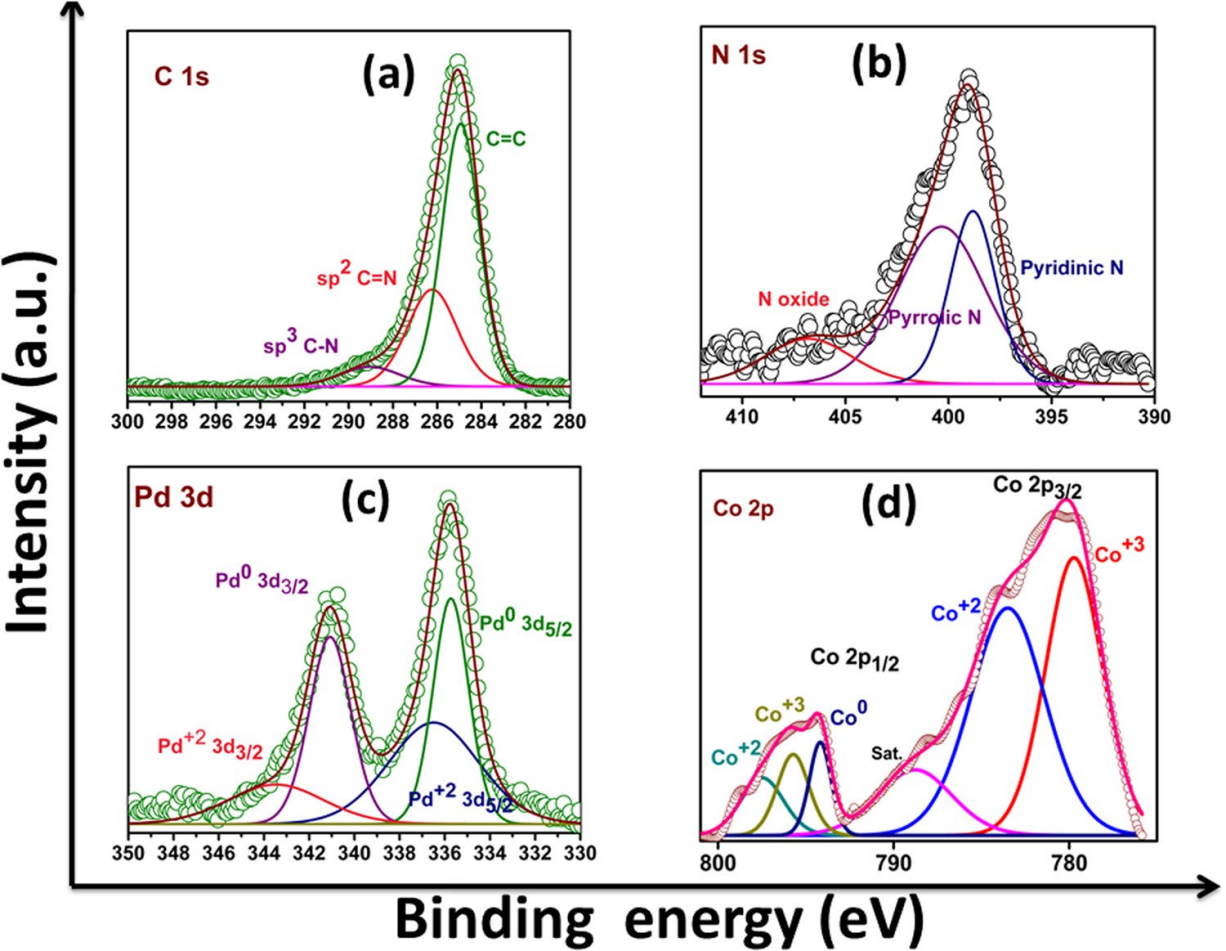
XPS spectra of (**a**) C 1s, (**b**) N 1s, (**c**) Pd 3d and (**d**) Co 2p orbitals of Pd_3_Co/NG.

X-ray photoelectron spectroscopy (XPS) is used to confirm the chemical composition of the sample Pd_3_Co/NG and to ensure nitrogen doping. Fig. S3 shows the XPS survey spectrum of Pd_3_Co/NG confirming the presence of carbon, nitrogen, palladium and cobalt. Fig. 3(a) shows the XPS spectrum of C 1s orbital. The deconvoluted spectrum of C 1s shows a prominent peak around 284.9 eV corresponding to sp^2^ C=C bonding. The other two peaks around 286.17 eV and 288.79 eV corresponds to the sp^2^ C=N and sp^3^ C-N bonding^33^. Fig. 3(b) shows the deconvoluted spectrum of N 1s. Three peaks around 398.82 eV, 400.28 eV and 406.62 eV corresponds to the pyridinic N, pyrolic N, and N-oxide respectively^27,34^. From XPS analysis, the amount of nitrogen atoms doped in the reduced graphene oxide structure was obtained as 1.268 atomic %. Fig. 3(c) shows the XPS spectrum of Pd 3d for Pd_3_Co/NG. It is deconvoluted into four peaks. The two intense peaks correspond to the metallic Pd (i.e. Pd^0^) and the other two peaks correspond to the +2 oxidation state of palladium (i.e. Pd^2+^). The peaks at 335.70 eV and 341.08 eV correspond to the Pd^0^ 3d_5/2_ and Pd^0^ 3d_3/2_ respectively. The +2 oxidation state of palladium (i.e. Pd^2+^) is due to the formation of Pd-O bond or Pd-N bond^35^ and the peaks corresponding to it is centered at 336.50 eV (Pd^+2^ 3d_5/2_) and at 343.48 eV (Pd^+2^ 3d_3/2_)^36^. Fig. 3(d) shows the deconvoluted spectrum of Co 2p for Pd_3_Co/NG. The two intense peaks at 780.11 eV and 794.73 eV correspond to the Co 2p_3/2_ and Co 2p_1/2_ respectively^37–39^. A shake-up satellite peak at 788.76 eV indicates the presence of Co_3_O_4_ which is denoted as Sat^40^. The atomic ratio of Pd^+2^/Pd^0^ and Pd/Co obtained from high resolution XPS spectra of Pd 3d and Co 2p was found to be 0.82 and 3.4 respectively.

**Figure 4:**
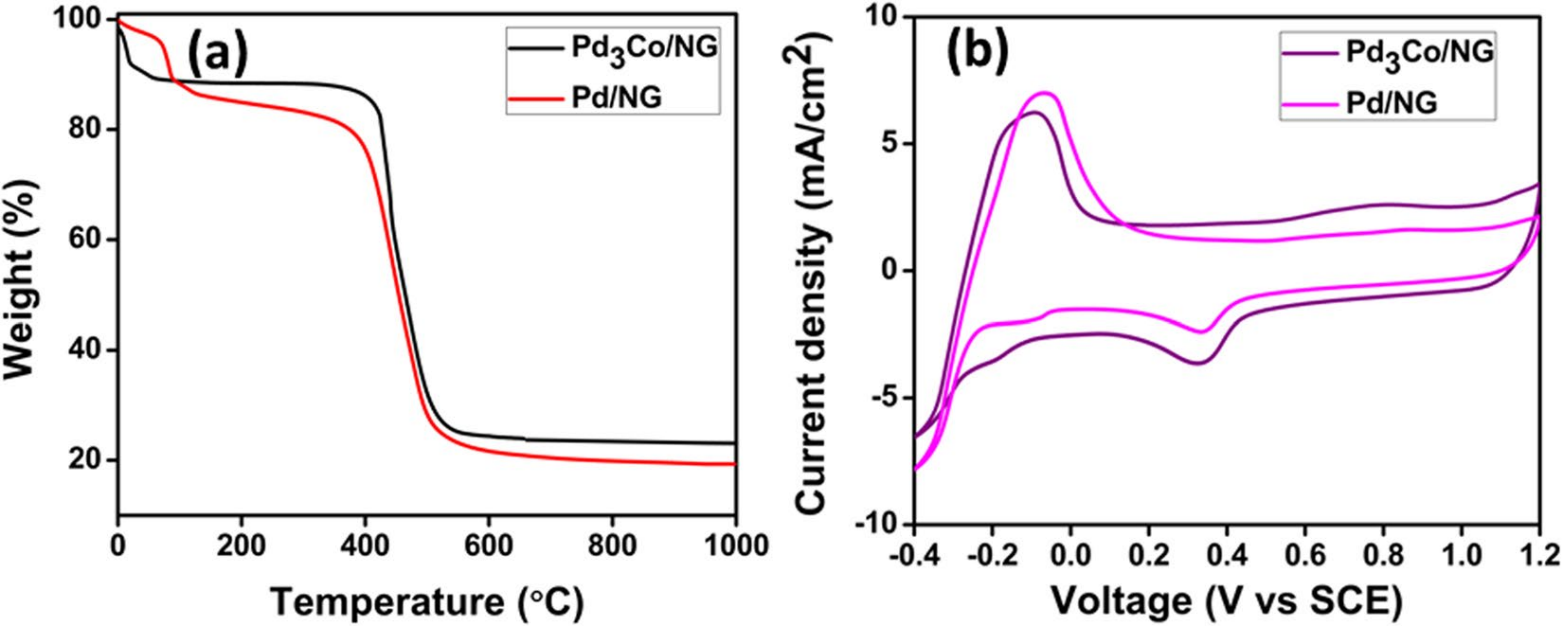
(**a**) TGA analysis of Pd_3_Co/NG and Pd/NG and (**b**) Cyclic voltammogram of Pd_3_Co/NG and Pd/NG.

Fig. 4(a) represents the Thermogravimetric analysis (TGA) profile of Pd_3_Co/NG and Pd/NG. The sample was heated from room temperature to 1000 °C in zero air atmosphere with a heating rate of 20 °C/min. The TGA profile shows a weight loss in the temperature range from 75 °C to 120 °C due to the loss of physisorbed residual water content present in the sample^41^. In the temperature range around 400 °C to 550 °C, another major weight loss was observed due to the decomposition of carbon in presence of air. Above 550 °C, the TGA profile was almost constant till 1000 °C without any weight loss, which confirms 23 wt% metal loading in Pd_3_Co/NG sample^42^ and about 20 wt% metal loading in Pd/NG.

Cyclic voltammograms (CV) were recorded in the potential range −0.4 V to 1.2 V vs saturated calomel electrode (SCE) at a scan rate of 50 mV/s in nitrogen saturated 0.5 M H_2_SO_4_ electrolyte for the samples Pd_3_Co/NG and Pd/NG. In the CV curve shown in Fig. 4(b), the peaks in the potential range from −0.25 V to 0.0 V are due to the hydrogen adsorption and desorption on the palladium surface. The broad peak above the potential 0.5 V during the anodic scan was due to the oxidation of palladium, whereas the peak obtained in the cathodic scan in the potential range from 0.45 V to 0.2 V was due to the reduction of palladium oxide to palladium metal. The electrochemical surface area (ECSA) for palladium-based catalyst is calculated using the formula,

1 $$ECSA=\frac{{Q}_{H}}{0.420\times [Pd]}$$

The coulombic charge Q_H_ for the palladium catalyst was calculated using the reduction peak of chemisorbed oxygen rather than using hydrogen adsorption/desorption peak in platinum-based electrocatalysts because Pd is basically a hydrogen storage element and it absorbs some amount of hydrogen making it difficult to calculate the exact coulombic charge using hydrogen adsorption/desorption peak^43,44^. [Pd] in the Equation (1) is the catalyst metal loading in the electrocatalyst and 0.420 mC cm^−2^ is the charge required for the full coverage of the Pd surface by monolayer of oxygen. The calculated ECSA using the Equation (1) was 24.6 m^2^ g^−1^ for Pd/NG and 39.8 m^2^ g^−1^ for Pd_3_Co/NG. The more ECSA of Pd_3_Co/NG is due to the formation of more active sites due to alloying. Fig. S4(a) shows the comparison of CV for Pd_3_Co/NG and palladium-cobalt alloy supported on reduced graphene oxide (Pd_3_Co/G). The ECSA calculated for Pd_3_Co/G was 32.2 m^2^ g^−1^. The better ECSA of Pd_3_Co/NG compared to that of Pd_3_Co/G might be attributed to the uniform and good dispersion of catalyst nanoparticles on graphene network due to the incorporation of nitrogen atoms.

**Figure 5:**
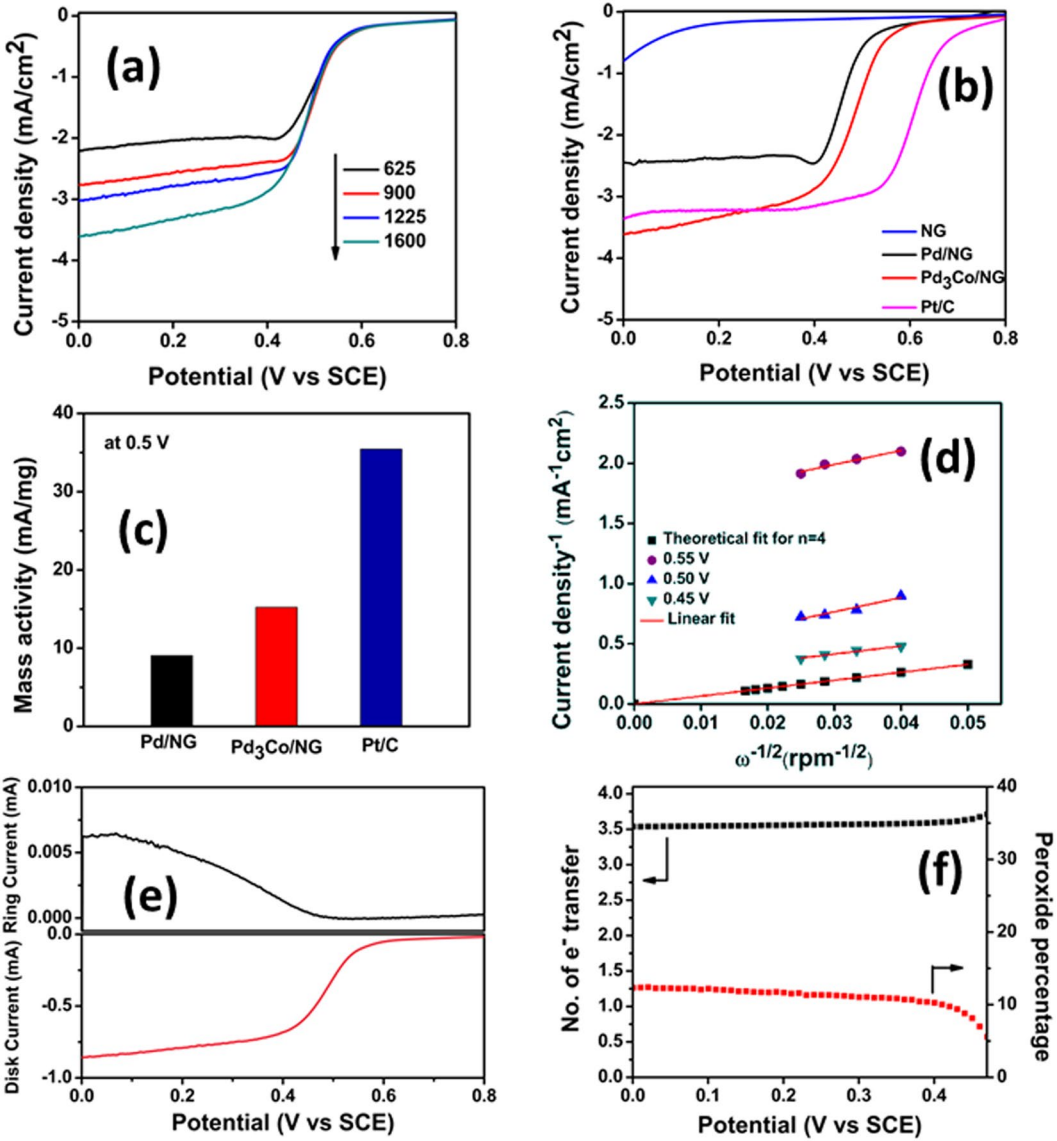
(**a**) LSV curve of Pd_3_Co/NG at different electrode rotation speed, (**b**) LSV curve of NG, commercial Pt/C, Pd_3_Co/NG and Pd/NG at 1600 rpm speed, (**c**) The mass activity of commercial Pt/C, Pd_3_Co/NG and Pd/NG at 0.5 V, (**d**) Koutecky-Levich plot of Pd_3_Co/NG, (**e**) RRDE curve of Pd_3_Co/NG at 1600 rpm and (**f**) Percentage of hydrogen peroxide produced and electron transfer number during ORR.

The ORR performance of the prepared electrocatalyst was investigated using rotating ring-disk electrode (RRDE) technique. Fig. 5(a) shows the linear sweep voltammetry (LSV) curve of Pd_3_Co/NG recorded at a scan rate of 10 mV s^−1^ at different rotational speed in oxygen saturated 0.5 M H_2_SO_4_ electrolyte solution at room temperature. From the Fig. 5(a), it is clear that the diffusion-limiting current increases with increase in rotational speed. With increase in electrode rotation, the diffusion of oxygen towards the electrode surface increases which in turn results in high oxygen reduction current^45–48^. Fig. 5(b) is the comparison of LSV curve for nitrogen-doped reduced graphene oxide (NG), Pd_3_Co/NG, Pd/NG and commercial Pt/C at 1600 rpm speed. It was observed that the onset potential of Pd_3_Co/NG was 607 mV vs SCE which is higher compared to that of Pd/NG having onset potential of 589 mV vs SCE. Also, the half-wave potential of Pd_3_Co/NG and Pd/NG was observed to be 490 mV and 469 mV vs SCE respectively. Additionally, the diffusion-limiting current density was observed to be high for Pd_3_Co/NG. Thus, the positive shift of the potential, high half-wave potential and high oxygen reduction current for Pd_3_Co/NG compared to Pd/NG confirmed higher ORR activity of Pd_3_Co/NG. Additionally, the LSV curve of NG shows an onset potential of 130 mV, though value is very less; it shows the contribution of NG to ORR activity. Thus, in comparison with NG, the composites of Pd_3_Co/NG and Pd/NG show better catalytic activities, indicating the major contribution of catalyst nanoparticles towards ORR activity than NG alone^27^. Further, the comparative LSV curve of commercial Pt/C shows the better catalytic activity of Pt-based electrocatalyst than Pd-based electrocatalysts. The mass activity of Pd_3_Co/NG, Pd/NG and commercial Pt/C at 0.5 V vs SCE was calculated based on the mass of the metallic catalyst and shown in Fig. 5(c). It was found that the mass activity of Pd_3_Co/NG was 1.6 times more than that of Pd/NG. The kinetic parameters derived from the polarization curve are tabulated in Table S2. Additionally, LSV of Pd_3_Co/G was recorded at 1600 rpm and has shown in the Fig. S4(b). It was observed that the calculated onset potential of Pd_3_Co/G (592 mV) was less compared to Pd_3_Co/NG, which further confirms the better ORR activity of the electrocatalyst with nitrogen doping.

The kinetic parameters related to ORR performance can be found out using Koutecky-Levich (K-L) plot using K-L Equation (2).

2 $$\frac{1}{j}=\frac{1}{{j}_{k}}+\frac{1}{{j}_{d}}=\frac{1}{nFk{C}_{{O}_{2}}}+\frac{1}{B{\omega }^{1/2}}$$

where *j* is the measured current density, *j*_*k*_ and *j*_d_ are the kinetic and diffusion current densities respectively. ω is the electrode rotation speed expressed in rpm and k is the rate constant^46,47^.

3 $$B=0.2nF{C}_{{O}_{2}}{D}_{{O}_{2}}{}^{2/3}{\nu }^{-1/6}$$

where n is the number of electrons transferred in ORR per oxygen molecule, F is the Faraday constant (96486 C mol^−1^), ${D}_{{O}_{2}}$ is the diffusion coefficient of oxygen in electrolyte (1.4 × 10^−5^ cm^2^ s^−1^), ν is the kinematic viscosity of the electrolyte (1 × 10^−2^ cm s^−1^) and ${C}_{{O}_{2}}$ is the concentration of oxygen in the electrolyte (1.1 × 10^−6^ mol cm^−3^)^47,48^. Fig. 5(d) shows the K-L lines of Pd_3_Co/NG at different potentials along with the theoretical line for four electron (n = 4) process. It can be seen that the experimental K-L lines are almost parallel to each other as well as to the theoretical n = 4 K-L line^48^. In addition to that, the number of electrons transferred per oxygen molecule during ORR, which can be obtained from the slope of the experimental K-L lines was found to be approximately 3.7. This suggests the ORR catalyzed by Pd_3_Co/NG was via nearly four-electron transfer mechanism.

For further investigation of ORR pathway, the RRDE technique was used. Fig. 5(e) shows the RRDE curve of Pd_3_Co/NG at 1600 rpm with 10 mV/s scan rate. The ring potential used was 1.0 V in order to oxidize the H_2_O_2_ produced on the disk. From RRDE data, the amount of hydrogen peroxide (H_2_O_2_) produced and the electron transfer number (n) during the ORR mechanism can be calculated. These parameters were calculated using the Equations (4) and (5).

4 $$ \% {H}_{2}{O}_{2}=\frac{200{i}_{r}/N}{{i}_{d}+{i}_{r}/N}$$

5 $$n=\frac{4{i}_{d}}{{i}_{d}+{i}_{r}/N}$$

where i_r_ is the ring current, i_d_ is the disk current and N is the collection efficiency (0.37)^49^.

Fig. 5(f) shows the calculated electron transfer number and the yield of hydrogen peroxide by Pd_3_Co/NG. The number of electrons transferred during the ORR mechanism was calculated to vary from 3.55 to 3.72 in the potential range 0 V to 0.48 V, which is almost equal to the value, obtained from the K-L plot. The percentage of peroxide produced varies from 6% to 12% in the same potential range mentioned above. These results show the ORR process catalyzed by Pd_3_Co/NG follows nearly four-electron pathway.

Electrochemical impedance spectroscopic (EIS) studies were carried out with the Pd_3_Co electrocatalyst with and without nitrogen doping. Fig. S5 shows the Nyquist plot of Pd_3_Co/NG and Pd_3_Co/G. In fuel cells, at higher potentials, the polarization resistance is dominated by the charge-transfer process and at lower potentials it is dominated by the mass-transfer process^50^. The inset in the figure shows the schematic representation of the equivalent circuit for EIS of the electrocatalyst, where R_1_ is the electrolyte resistance, R_2_ is the resistance due to the contact resistance, R_3_ is the charge transfer resistance and CPE is the constant phase element. After circuit fitting, the values of the circuit elements are tabulated and included as Table S3. Fitted results reveal that the charge-transfer resistance of Pd_3_Co/NG is much less compared to the electrocatalyst Pd_3_Co/G. The less charge-transfer resistance of Pd_3_Co/NG shows the good catalytic activity. This may be attributed to the availability of more active sites with the incorporation of nitrogen atoms on graphene network and due to the uniform distribution of Pd_3_Co nanoparticles over the surface of NG with less agglomeration^4^.

**Figure 6:**
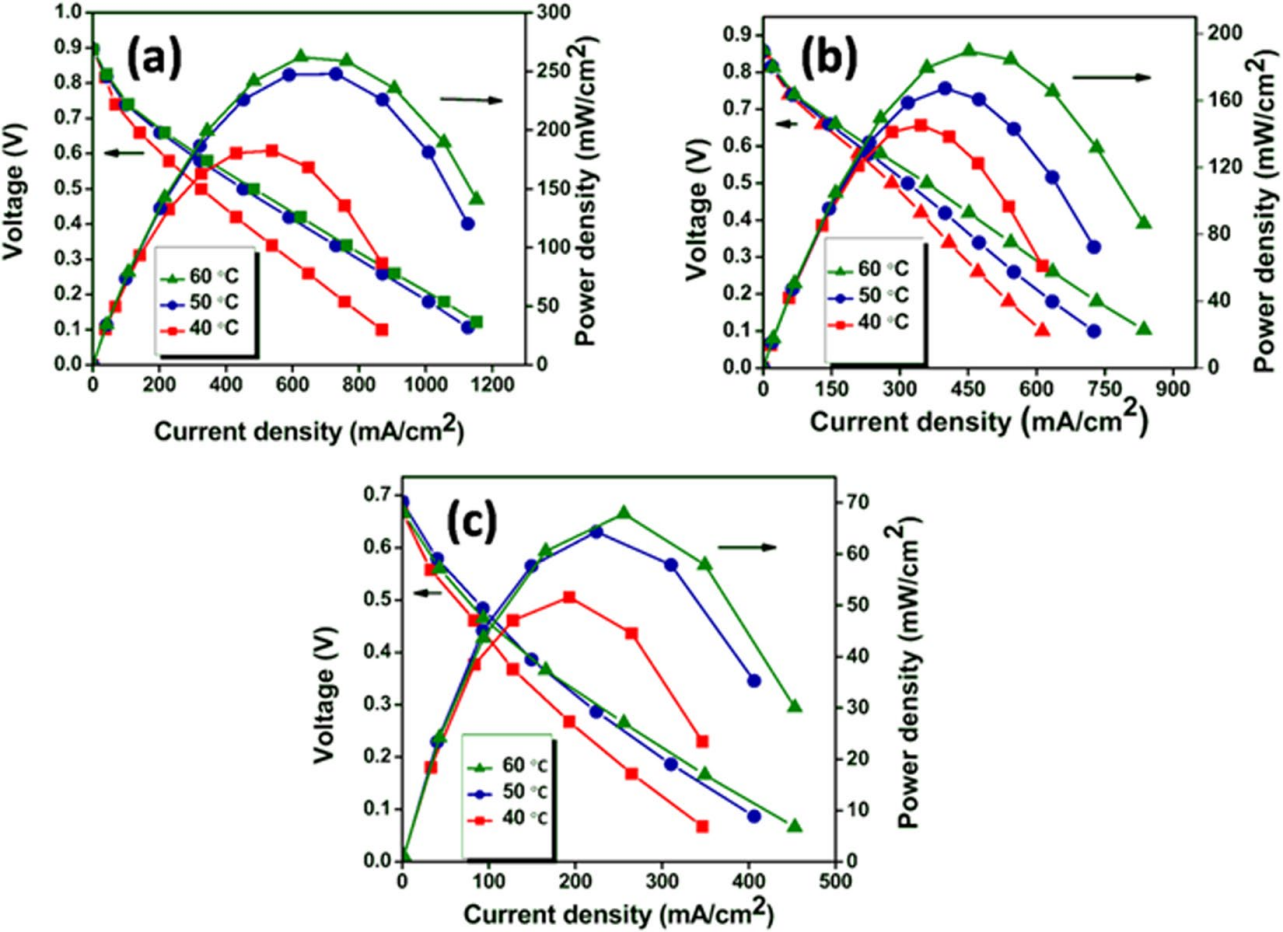
Polarization curves of (**a**) MEA 1, (**b**) MEA 2 and (**c**) MEA 3 at 40 °C, 50 °C, and 60 °C temperature.

Polarization studies were performed by preparing membrane electrode assembly (MEA) using Pd_3_Co/NG as anode electrocatalyst with catalyst loading of 0.5 mg/cm^2^ and commercial Pt/C as cathode electrocatalyst with catalyst loading of 0.1 mg/cm^2^. The above MEA is labeled as MEA 1. The measurements were carried out by reversing anode and cathode, which is labeled as MEA 2. Finally, the MEA was prepared using Pd_3_Co/NG (0.5 mg/cm^2^) on both anode and cathode electrocatalyst and labeled as MEA 3. For comparison, three more MEAs with Pd/NG as anode electrocatalyst, cathode electrocatalyst and at both anode and cathode were prepared and labeled as MEA 4, MEA 5 and MEA 6 respectively. Similarly, again three more MEAs with Pd_3_Co/G as anode electrocatalyst, cathode electrocatalyst and at both anode and cathode were prepared and labeled as MEA 7, MEA 8 and MEA 9 respectively. The polarization measurements were recorded at 40 °C, 50 °C and 60 °C. Fig. 6(a–c) shows the polarization curve of MEA 1, MEA 2 and MEA 3, respectively. Fig. S6(a–c) show the polarization curve of MEA 4, MEA 5 and MEA 6, respectively. Similarly Fig. S7(a–c) show the polarization curve of MEA 7, MEA 8 and MEA 9 respectively. Before the polarization measurement, MEAs were activated between open circuit potential (OCP) and 0.1 V. All polarization studies were carried out without backpressure.

In general, for all prepared MEAs, a considerable improvement in fuel cell performance was observed with increase in temperature. This was mainly due to the enhancement of hydrogen oxidation and oxygen reduction reaction kinetics with the rise in temperature. The MEA 1 with Pd_3_Co/NG used as anode electrocatalyst and Pt/C used as cathode electrocatalyst showed a maximum power density of 262 mW/cm^2^ at 60 °C. The MEA 2 showed a maximum power density of 189 mW/cm^2^ at 60 °C. In MEA 2, Pd_3_Co/NG was used as cathode electrocatalyst and the performance was much better than Pt-based cathode electrocatalyst reported earlier, by keeping Pt/C at anode. For instance, Seger *et al*. demonstrated a maximum power density of 161 mW/cm^2^ at 60 °C with partially reduced GO-Pt based electrocatalyst at cathode^51^. It is clear from the polarization measurements that Pd_3_Co/NG shows good HOR and ORR activity. The good catalytic activity of Pd_3_Co/NG composites can be due to the synergistic catalytic effect of nitrogen doped reduced graphene oxide network and the Pd-Co alloy nanoparticles. The good HOR and ORR performance was due to better adherence between the catalyst nanoparticles and the reduced graphene oxide through nitrogen doping. When nitrogen is incorporated into graphene network, the doped nitrogen acts as a connection link between the catalyst nanoparticles and the support material which results in low electrochemical impedance and strengthen the interaction between them^27^. Thus, nitrogen provides better pathway for the transport of electrons, which helps in the enhancement of the catalytic activity. In addition to that, nitrogen doping provides changes in the charge distribution of the carbon atoms in graphene network, in such a way that, it favors in transfer of charge from carbon to the adsorbed oxygen molecule which in turn helps in the weakening of O-O bond and promotes the dissociation results in improved ORR activity^21^. Moreover, it was reported in literature that nitrogen-doped graphene promotes the oxygen dissociation mechanism, by reducing the energy barrier for the dissociation of oxygen which is more sluggish reaction in fuel cell^52^. In addition to that, the alloying effect also plays an important role in enhancing the HOR and ORR performance. The bimetallic catalyst Pd-Co exhibits better catalytic activity by combining the catalytic properties of individual elements in a collective way which leads to more active surface than Pd alone^9^. The MEA 3 prepared by using Pd_3_Co/NG as cathode and anode electrocatalyst showed maximum power density of 68 mW/cm^2^ at 60 °C, which was an appreciable power density in the absence of Pt. This was the first attempt using Pd_3_Co/NG as the electrocatalyst at anode as well as cathode without using Pt. Similarly MEA 4, MEA 5 and MEA 6 showed maximum power density of 195 mW/cm^2^, 158 mW/cm^2^ and 35 mW/cm^2^ respectively. Moreover, MEA 7, MEA 8 and MEA 9 displayed maximum power density of 232 mW/cm^2^, 129 mW/cm^2^ and 42 mW/cm^2^ for Pd_3_Co/G, respectively. It can be seen that Pd_3_Co/NG exhibits more catalytic performance than Pd/NG and Pd_3_Co/G due to the synergetic effect of nitrogen doping and alloying resulting into the formation of more active surface, which was supported by cyclic voltammetry analysis. The maximum power density obtained for different MEAs at 60 °C temperature is listed in Table 1.

**Table 1:** Maximum power density obtained for different MEA’s at 60 °C temperature with Pt/C loading of 0.1 mg/cm^2^ and Pd_3_Co/NG (Pd/NG, Pd_3_Co/G) loading of 0.5 mg/cm^2^.

Sl.No.	MEA	Anode	Cathode	Maximum Power density at 60 °C (mW/cm^2^)
1.	MEA 1	Pd_3_Co/NG	Pt/C	262
2.	MEA 2	Pt/C	Pd_3_Co/NG	189
3.	MEA 3	**Pd**_**3**_**Co**/**NG**	**Pd**_**3**_**Co**/**NG**	68
4.	MEA 4	Pd/NG	Pt/C	195
5.	MEA 5	Pt/C	Pd/NG	158
6.	MEA 6	Pd/NG	Pd/NG	35
7.	MEA 7	Pd_3_Co/G	Pt/C	232
8.	MEA 8	Pt/C	Pd_3_Co/G	129
9.	MEA 9	Pd_3_Co/G	Pd_3_Co/G	42

## Conclusion

In summary, we have successfully synthesized Pd_3_Co/NG by a simple and single-step synthesis route. Uniform dispersion of Pd-Co alloy nanoparticles has been achieved on NG through this method by incorporating nitrogen in graphene lattice and used as anode electrocatalyst, cathode electrocatalyst and as both in PEMFC. Pd_3_Co/NG exhibits a good single cell performance due to the influence of nitrogen doping and the alloying effect of bimetallic catalysts. As anode and cathode electrocatalyst, Pd_3_Co/NG in combination with Pt/C, reveals a maximum power density of 262 mW/cm^2^ and 189 mW/cm^2^ respectively at 60 °C without any backpressure. RRDE results indicate that the ORR process catalyzed by Pd_3_Co/NG follows nearly a four-electron mechanism. When used as anode as well as cathode electrocatalyst simultaneously with individual loading of 0.5 mg/cm^2^, the full cell with Pd_3_Co/NG yields a maximum power density of 68 mW/cm^2^ at 60 °C without any backpressure. Thus the results suggest that Pt-free Pd_3_Co/NG holds a great application potential as a promising electrocatalyst in PEMFC due to the advantages of facile preparation and outstanding catalytic performance.

## Materials and Methods

### Materials

Graphite (99.9%) was obtained from Sigma Aldrich. Sodium nitrate (NaNO_3_, 99.5%), potassium permanganate (KMnO_4_, 99.5%), concentrated sulphuric acid (H_2_SO_4_, 98%) were used for the synthesis of GO were procured from Rankem Chemicals. Hydrogen peroxide (H_2_O_2_, 30%) was purchased from SD Fine-Chem Ltd, India. Melamine (C_3_H_6_N_6_) was purchased from Himedia Laboratory Pvt. Ltd India. Palladium (II) chloride and cobalt (II) chloride hexahydrate were procured from Sigma Aldrich. Commercial Pt/C (Tanaka) and deionized (DI) water were used in the experiment.

### Material Synthesis

Graphitic oxide was synthesized by Hummers method^53^. The Hummers method is explained briefly as follows. Graphite powder (2 g) was added into the beaker containing 46 ml of conc. H_2_SO_4_ in an ice bath. The oxidizing agents like NaNO_3_ (1 g) and KMnO_4_ (6 g) were added very slowly into it. The above mixture was allowed to stir for 15 min. The beaker was removed from the ice bath and kept it for stirring for another 45 min till it reaches the room temperature. Then 92 ml of DI water was added in drops using dropper. To the above mixture, 282 ml of warm DI water was added. Finally, 12 ml of hydrogen peroxide was added into the beaker. The final suspension was filtered and dried at 60 °C in vacuum oven to obtain GO.

Single-step reduction of Pd_3_Co/NG was prepared by the procedure as follows. Melamine was used as the nitrogen source. Required amount of GO and melamine (2:1 ratio) were dispersed in de-ionized water and ultrasonicated for 15 min to obtain the uniform mixture. To the above suspension, 1 wt% solution of palladium (II) chloride (PdCl_2_) and cobalt (II) chloride hexahydrate (CoCl_2_.6H_2_O) were added dropwise. The above suspension was allowed to stir for six hours. The above mixture was dried in vacuum oven at 60 °C. The dried sample was taken in a quartz boat and was placed at the centre of the tubular furnace in a quartz tube. The furnace was flushed with Argon (Ar) gas for 10 min. The furnace temperature was raised to 500 °C and the temperature was maintained for 30 minutes under hydrogen flow. Then the sample was heated up to 700 °C and the temperature was maintained for 30 min under Ar flow. Then the furnace was allowed to cool to room temperature and the resulting sample was labeled as Pd_3_Co/NG. Similarly, Pd/NG and Pd_3_Co/G was prepared for comparison in the above-mentioned method.

### Characterizations

The powder X-ray diffraction (XRD) were obtained using Rigaku X-ray diffractometer with X-ray source of wavelength λ = 0.15406 nm at 40 kV voltage and 30 mA current. The XRD data were recorded in the range of 2θ values from 5° to 90° in step size of 0.016°. The Raman spectra were recorded with WITec alpha 300 Confocal Raman spectrometer using Nd:YAG laser as excitation source of wavelength 532 nm. The morphology of the synthesized sample was studied using field emission scanning electron microscopy (FESEM, FEI Quanta 200) and transmission electron microscopy (TEM, Technai G-20). The sample preparation for TEM was done by ultrasonicating the sample in ethanol and drop casted over carbon coated 200 mesh copper grid. Thermo gravimetric analysis (TGA) of the synthesized sample was carried out using SDTQ600 TA instruments from room temperature to 1000 °C with a heating rate of 20 °C min^−1^ in zero air atmosphere. The oxidation state of the elements of the sample was confirmed by X-ray photoelectron spectroscopy carried out using SPECS instrument and PHOIBOS 100MCD as the analyzer.

### Electrochemical Measurements

Electrochemical characterization studies were carried out in 0.5 M H_2_SO_4_ electrolyte. Cyclic voltammetry (CV) measurements in nitrogen atmosphere were carried out in BioLogic science instruments using an electrochemical cell, which consists of a reference electrode, a counter electrode and a working electrode. Platinum wire was used as counter electrode and saturated calomel electrode (SCE) as reference electrode. The working electrode used was a glassy carbon disk on a Teflon cylinder into which electrocatalyst slurry was drop casted. Before drop casting the slurry, glassy carbon electrode was polished with 0.05 μm alumina paste. The electrocatalyst slurry was prepared by ultrasonicating 3.5 mg of sample in 170 μl of DI water and 6 μl of 5 wt% Nafion solution for 30 min. After that, the required amount of slurry was drop casted on the glassy carbon electrode and allowed to dry at room temperature. The RRDE with glassy carbon disk and a Pt ring was used as the working electrode to investigate the ORR activity of the prepared catalyst using BioLogic science instruments. The catalyst preparation method for RRDE was same as the preparation for CV measurement.

The single cell measurement was carried out with the preparation of membrane electrode assembly (MEA). The MEA was fabricated according to the method described below. The MEA was prepared by sandwiching a Nafion membrane between the anode and the cathode. The electrocatalysts were prepared by brush coating the electrocatalyst ink over the gas diffusion layer (GDL), one-sided teflonized carbon cloth (Nickunj Eximp Entp Pvt Ltd, India). The electrocatalyst ink was prepared by ultrasonicating the required amount of electrocatalyst in the mixture of deionized (DI) water, isopropyl alcohol and 5 wt% Nafion solution. The effective electrode area was 11.56 cm^2^. The Nafion 212 membrane was pretreated before using in MEA fabrication. The membrane was first heated with 3% H_2_O_2_ for 1 h at 80 °C. After reaching room temperature, the membrane was washed thoroughly with DI water. Then, it was heated with 1 M H_2_SO_4_ at 80 °C for 1 h and washed with DI water. Finally, the MEA was prepared by sandwiching Nafion 212 membrane between the anode and the cathode by hot pressing at 130 °C, 1 ton pressure for 4 min.

The single cell measurement was carried out in TELEDYNE MEDUSA^TM^ RD fuel cell test station. Graphite flow field plates with serpentine geometry were used for assembling the MEA. To control the hydrogen and oxygen gases in anode and cathode side the mass flow controllers were used. The humidifiers were provided to maintain humidification for the incoming gases to the electrodes. The humidified gases were fed to the electrodes with a flow rate of 50 sccm.

## Electronic supplementary material

Supplementary Information
