# Supplementary material for: High-performance Platinum-free oxygen reduction reaction and hydrogen oxidation reaction catalyst in polymer electrolyte membrane fuel cell
Source: Sci Rep. 2018 Feb 26;8:3591. doi: 10.1038/s41598-018-22001-9 (PMC5827662; doi:10.1038/s41598-018-22001-9)
Supplement: Supplementary file 1 — Supplementary Information [file 41598_2018_22001_MOESM1_ESM.doc]

**Supporting Information**

**High-performance Platinum-free oxygen reduction reaction and hydrogen oxidation reaction catalyst in polymer electrolyte membrane fuel cell**

Priji Chandran, Arpita Ghosh and ^*^S. Ramaprabhu

Alternative Energy and Nanotechnology Laboratory (AENL), Nano-Functional Materials and Technology Centre (NFMTC), Department of Physics, Indian Institute of Technology Madras, Chennai, Tamil Nadu, 600036, India.

^*^Email: [ramp@iitm.ac.in](mailto:ramp@iitm.ac.in)

**
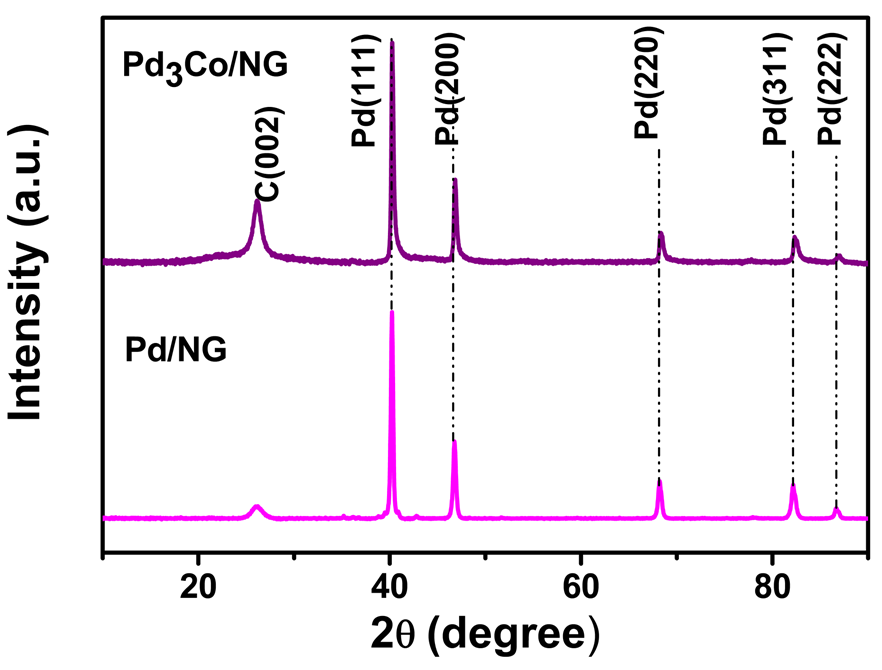
**

**Fig. S1: XRD pattern of Pd_3_Co/NG and Pd/NG.**


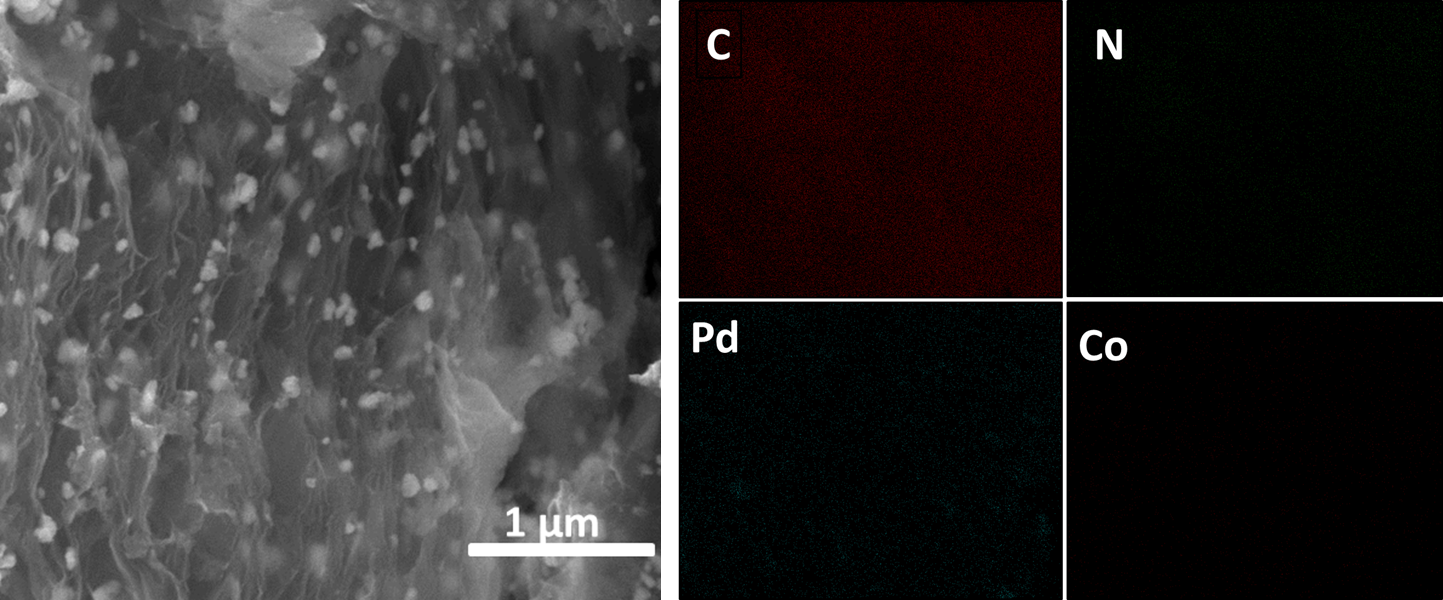


**Fig. S2: SEM image and Elemental mapping of the Pd_3_Co/NG.**


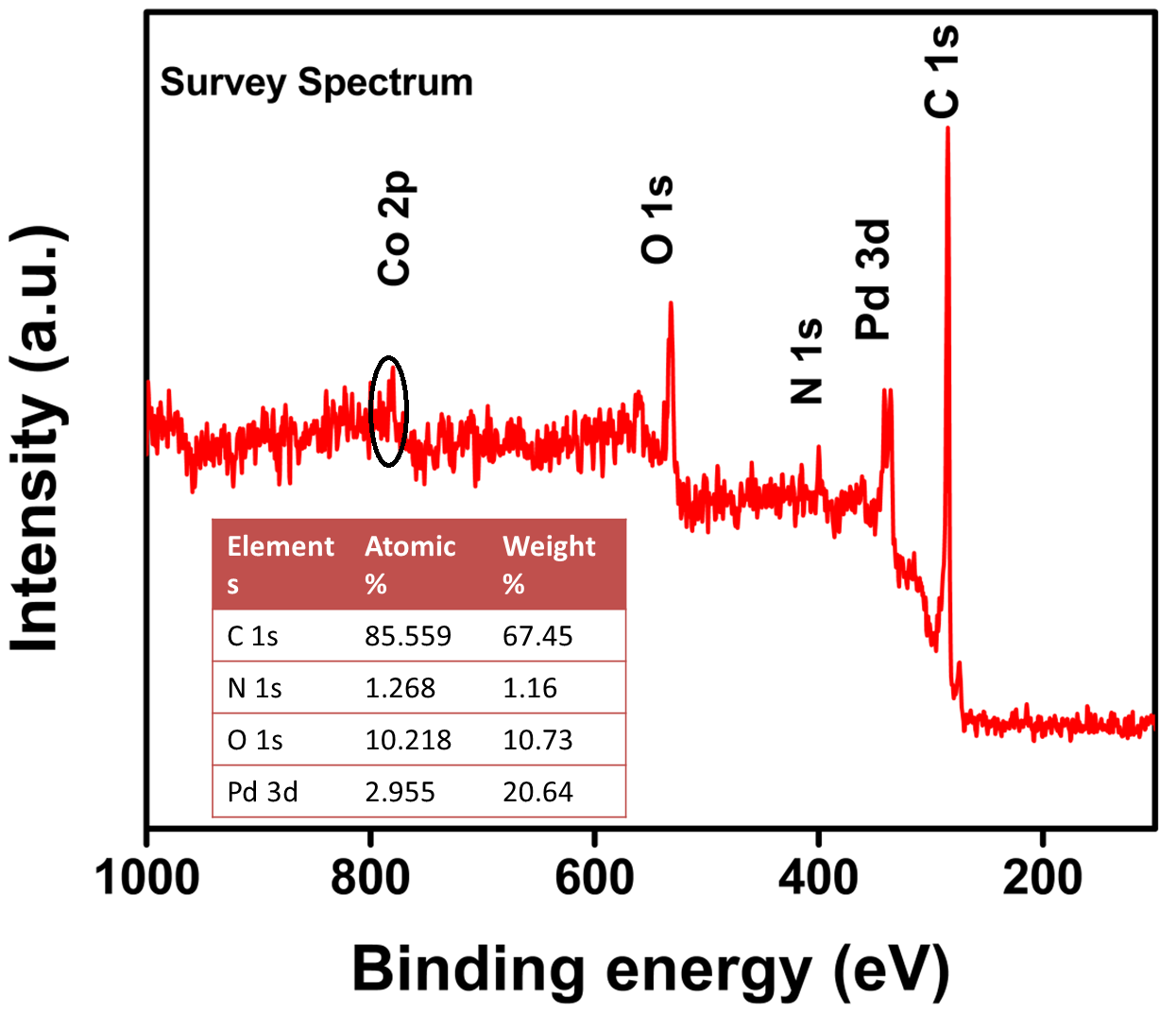


**Fig. S3: XPS survey spectrum of Pd_3_Co/NG**.


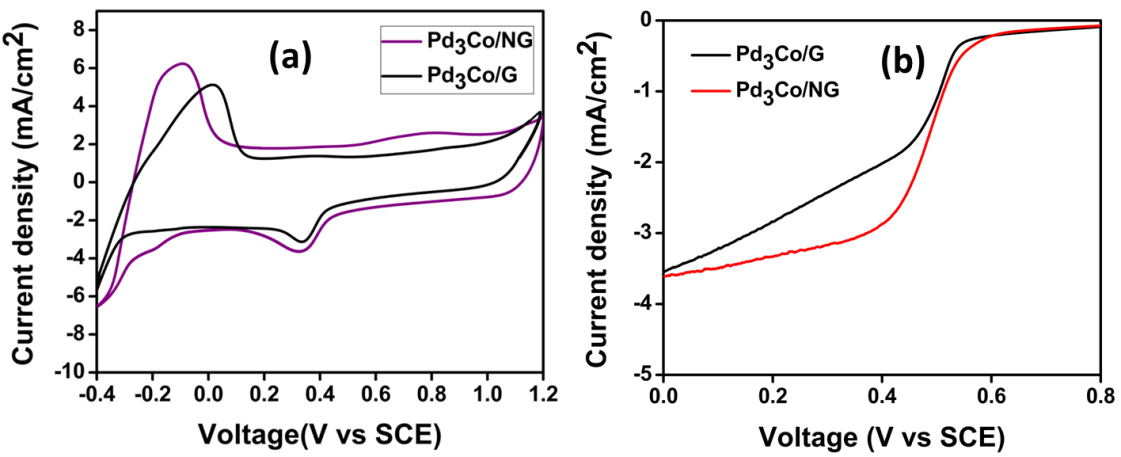


**Fig. S4: (a) Cyclic voltammogram of Pd_3_Co/NG and Pd_3_Co/G and (b) LSV curve of Pd_3_Co/NG and Pd_3_Co/G at 1600 rpm speed.**


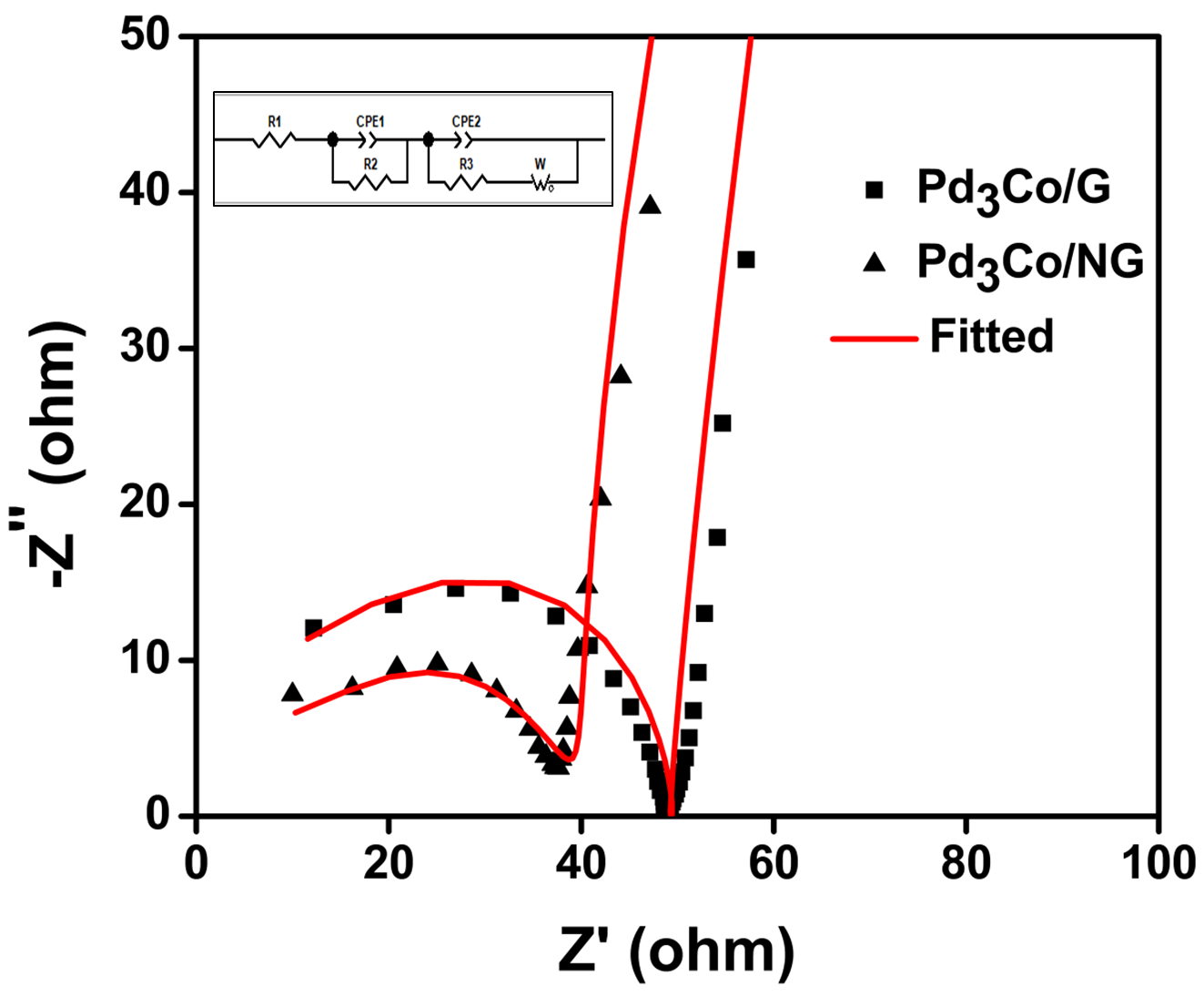


**Fig. S5: Electrochemical impedance spectra of Pd_3_Co/NG and Pd_3_Co/G.**


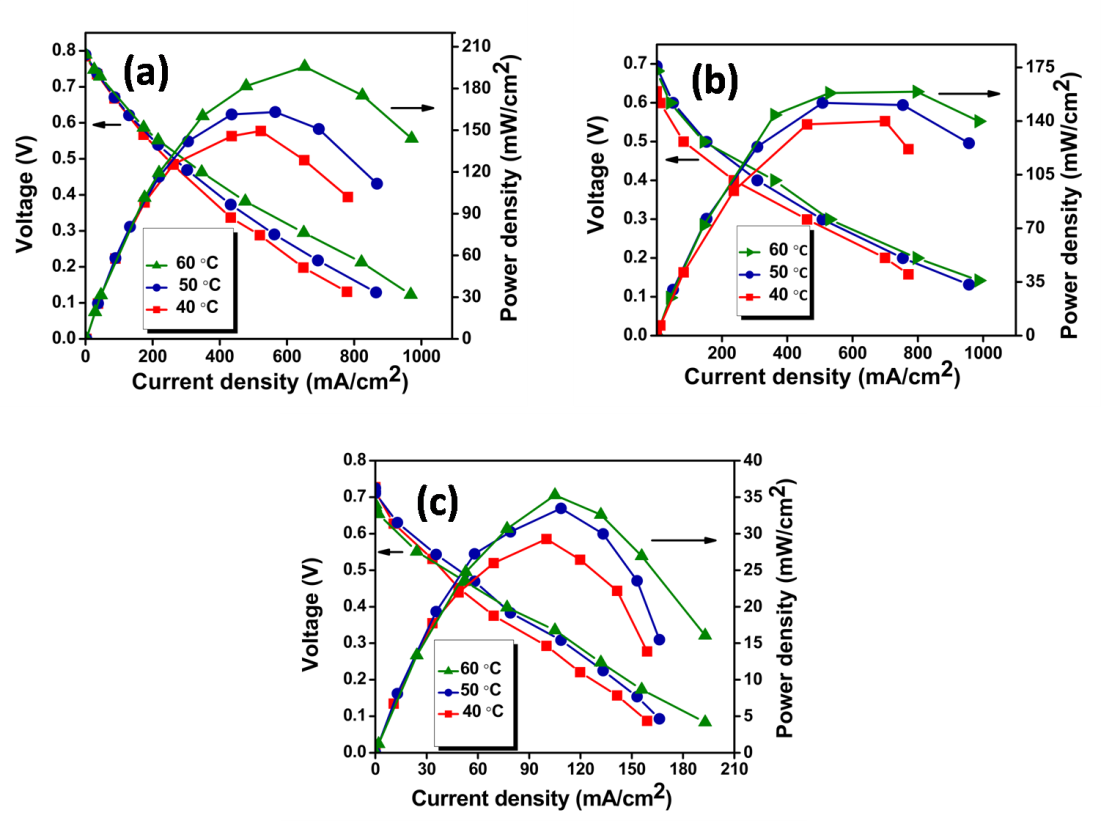


**Fig. S6: Polarization curves of (a) MEA 4, (b) MEA 5 and (c) MEA 6 at 40 ⁰C, 50 ⁰C, and 60 ⁰C temperature.**

**
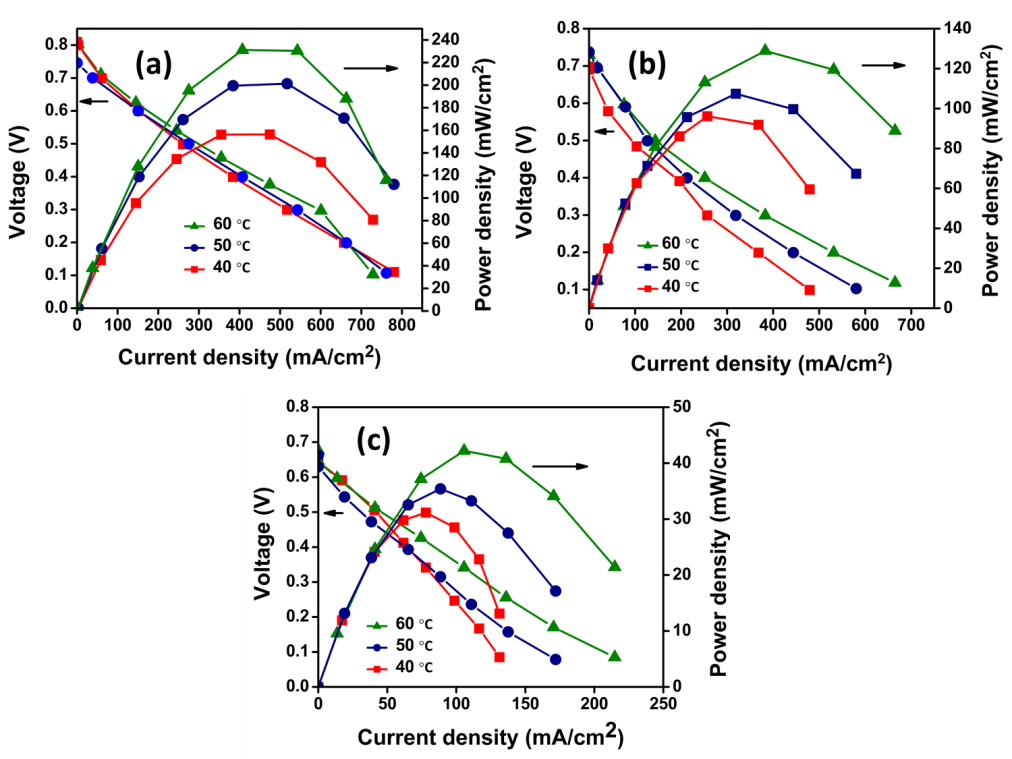
**

**Fig. S7: Polarization curves of (a) MEA 7, (b) MEA 8 and (c) MEA 9 at 40 ⁰C, 50 ⁰C, and 60 ⁰C temperature**.

Table S1: I _D_ / I _G_ ratio of the samples from Raman spectrum.

| Sl. No. | **Sample name** | **I _D_ / I _G_ ratio** |
| --- | --- | --- |
| 1. | Graphite | 0.21 |
| 2. | GO | 1.07 |
| 3. | Pd3Co/NG | 1.11 |

Table S2: Kinetic parameters derived from ORR polarization curve.

| **Factors of the ORR activity** | **Electrocatalyst**  **Pd_3_Co/NG** | **Electrocatalyst**  **Pd/NG** | **Commercial Pt/C** |
| --- | --- | --- | --- |
| Onset potential (mV vs SCE) | 607 | 589 | 741 |
| Half-wave potential (mV vs SCE) | 490 | 469 | 609 |
| Mass activity at 0.5 V vs SCE (mA mg^-1^) | 15.23 | 9.05 | 35.45 |

Table S3: Fitted results of EIS spectra

| **Sample** | **R_1_ (ohm)** | **R_2_ (ohm)** | **R_3_ (ohm)** |
| --- | --- | --- | --- |
| Pd_3_Co/NG | 5.058 | 5.57 | 5.902 |
| Pd_3_Co/G | 5.098 | 30.87 | 35.2 |
